# Relapsing and progressive MS: the sex-specific perspective

**DOI:** 10.1177/1756286420956495

**Published:** 2020-09-23

**Authors:** Paulus Stefan Rommer, David Ellenberger, Kerstin Hellwig, Judith Haas, Dieter Pöhlau, Alexander Stahmann, Uwe Klaus Zettl, on behalf of the Scientific Advisory Group of the German MS-Register by the German MS Society

**Affiliations:** Department of Neurology, Neuroimmunological Section, University of Rostock, Gehlsheimer Straße 20, Rostock, 18147, Germany; Department of Neurology, Medical University of Vienna, Spitalgasse 23, Vienna, 1090, Austria; German MS-Register by the German MS Society, MS Forschungs- und Projektentwicklungs-gGmbH, Hannover, Germany; Department of Neurology, Katholisches Klinikum Bochum, St. Josef Hospital, Ruhr University Bochum, Bochum, Germany; Centre for Multiple Sclerosis, Jewish Hospital Berlin, Berlin, Germany; Department of Neurology, German Red Cross-Kamillus-Clinic, Asbach, Germany; German MS-Register by the German MS Society, MS Forschungs- und Projektentwicklungs-gGmbH, Hannover, Germany; Department of Neurology, Neuroimmunological Section, University of Rostock, Rostock, Germany

**Keywords:** age of onset, multiple sclerosis, progressive MS, relapsing MS, sex ratio

## Abstract

**Background:**

Multiple sclerosis (MS) is an inflammatory and neurodegenerative disease whose aetiology is not fully understood. The female sex is clearly predominant, with a sex ratio between 2 and 3. In primary progressive MS the sex ratio almost balances out. Since the age at onset is higher for patients with progressive onset (POMS) than for relapsing onset (ROMS), it can be hypothesized that the age at onset is a decisive factor for the sex ratio.

**Methods:**

To address this aspect, we compare clinical and demographic data between females and males for the different disease courses within the population of the German MS Register by the German MS Society. Only patients with complete details in mandatory data items and a follow-up visit since 01. Jan 2018 were included.

**Results:**

A total of 18,728 patients were included in our analyses, revealing a female-to-male ratio of 2.6 (2.7 for patients with ROMS and 1.3 for POMS). The age at diagnosis is higher in patients with POMS (43.3 and 42.3 years for females and males *versus* 32.1 and 33.2 years, respectively). Females irrespective of disease course are statistically significantly more often affected by cognitive impairment (POMS: *p* = 0.013, ROMS: *p* = 0.001) and depression (POMS: *p* = 0.002, ROMS: 0.001) and suffer more often from pain (POMS and ROMS: *p* < 0.001). Fatigue is significantly more often seen in females with ROMS (*p* < 0.001) but not in POMS. Females with ROMS retire significantly (*p* < 0.001) earlier (42.8 *versus* 44.2 years) and to a greater extent than males (28 *versus* 24%). Disease progression was similar for women and men.

**Conclusion:**

Our analysis shows that clinical and demographic data differ more between disease courses than between men and women. For pain, depression and cognitive impairment the female sex is the decisive factor. Whether these factors are responsible for the earlier retirement of females with ROMS is not clear. Appropriate measures for optimization of symptomatic treatment as well as to promote employment should be taken.

## Introduction

Multiple sclerosis (MS) is an inflammatory and neurodegenerative disease that occurs primarily in young adults and whose aetiology is not fully understood.^1,2^ The clinical course of MS is very heterogeneous and a distinction can be made between relapsing and progressive courses.^3,4^ The age of onset is around 30 years in patients with relapsing onset (ROMS) and around 42 years in patients with progressive onset (POMS). In MS there is a significant predominance of the female sex compared with males,^1,5^ with a female-to-male ratio (sex ratio) between 2 and 3 that has been increasing over the last decades.^6,7^ The causes are not clear, but lifestyle changes and environmental interactions may have changed the risk of being affected by MS over recent years. Increased cigarette smoking, higher body mass index may have increased the risk of developing MS, while diet (especially consumption of fish) and outdoor activities may have reduced the risk. Changes in reproductive behaviour and hormonal changes also have an impact on the risk of developing MS. The latter includes taking contraceptives and the average later birth of the first child.^8–11^ These environmental factors are changing, are heterogeneously distributed in populations and may occur in mutually opposing ways. The exact role on MS is therefore difficult to determine.

The causes are not clear, but various sex-specific environmental interactions might have changed over time, such as cigarette smoking, diet (especially consumption of fish), urban lifestyles, outdoor activities, body mass index, hormone changes in women and reproductive behaviour, which might play a role on the risk of MS. An interesting observation is that the sex ratio almost balances out in POMS.^12,13^ Since the age at onset is higher in patients with POMS than in patients with ROMS, and since the sex ratio between POMS and ROMS is different, we want to examine to what extent the varying pathophysiology between both disease courses^14^ is reflected in the clinical spectrum of patients. To answer this question, we analyse the female-to-male ratio (sex ratio) for clinical and demographic data and for the various disease courses. On the one hand, whether the clinical data of women and men differ within the respective disease courses (comparison of women and men separately for ROMS and POMS) will be analysed, and on the other hand whether the data for the respective sex differ between the different disease courses (clinical data of women and men in direct comparison between ROMS and POMS).

## Patients and methods

The German MS Register (GMSR; Deutsches Multiple Sklerose Register) was established by the German MS Society (Deutsche Multiple Sklerose Gesellschaft, DMSG) in 2001 to provide a comprehensive insight into the status of people with MS (PwMS) in Germany.^15^ For the analysis presented here, data were extracted from the GMSR in March 2020. Only patients for whom data on the basic variables sex, date of birth, date of onset of the disease, and disease course at onset and symptoms were available and who had had a recent follow-up visit after 1 January 2018 were analysed. Data from the last visit are assessed. Descriptive statistics include frequencies and percentages for categorical data, means and standard deviations for metric data, and median and quartiles for ordinal data. A two-way analysis of variance was performed to compare both sexes, demographic data, symptoms and their interaction effects. For binary outcomes generalized linear models were used with logistic link function. To achieve robust inference additional matched analyses were carried out, in which each male with MS was 1:1-matched with a female with MS by year of birth, year of onset and disease course at onset, to avoid confounding. Data transformation and statistical analysis were performed using R 3.6.3 (R Foundation for Statistical Computing. Vienna, Austria).

Anonymized data will be made available on request by any qualified investigator under the terms of the registries’ usage and access guidelines and subject to informed consent of the patients.

The GMSR was registered with the German Register of Clinical Studies (DRKS; Deutsches Register Klinischer Studien, DRKS; No. DRKS00011257). Ethical approval for the registry and analysis was received by the IRB at the University Hospital of Würzburg (No. 142/12).

## Results

Data were from 21,119 patients who had no open queries and sufficient follow-up visits since 1 January 2018. Patients excluded either because of missing date of onset or because the disease course at onset was not definite totalled 2,391. Thus, a total of 18,728 patients were included in the subsequent analyses. Table 1 presents demographic data on the patients stratified by disease course at onset and sex.

**Table 1.** Demographic data of analysed patients.

	ROMS Females *n* = 12,819	ROMS Males *n* = 4,778	*p*-value (ROMS)	POMS Females *n* = 640	POMS Males *n* = 491	*p*-value (POMS)
Disease duration, mean (SD)	14.1 (10.1)	13.1 (9.6)	<0.001	15.1 (10.7)	13.8 (9.6)	0.034
Age at onset, mean (SD)	32.1 (10.3)	33.2 (10.3)	<0.001	43.3 (11.0)	42.3 (11.0)	0.11
Time to diagnosis, mean (SD)	1.7 (3.9)	1.5 (3.7)	0.088	2.9 (5.5)	2.8 (4.6)	0.83
EDSS, mean (SD)	2.9 (2.1)	3.1 (2.2)	<0.001	5.1 (2.0)	5.0 (1.8)	0.70
Current DMT, any type: yes/no, %	77%	80%	<0.001	42%	48%	0.052
Age retired	42.8 (9.56)	44.2 (9.29)	<0.001	49.4 (8.59)	48.7 (9.52)	0.49
Early retirement, %	28%	24%	<0.001	47%	43%	0.29

Early retirement = inability to work due to multiple sclerosis (MS).

DMT, disease modifying therapy; EDSS, Expanded Disability Status Scale; POMS, progressive onset of MS; ROMS, relapsing onset of MS.

**Figure 1.**
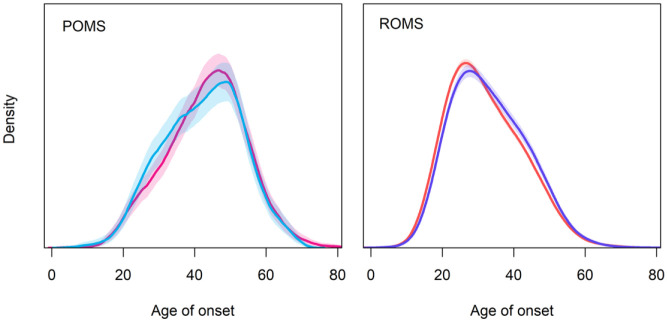
Age at onset for females (red) and males (blue) and progressive onset multiple sclerosis (POMS; left) and relapsing onset multiple sclerosis (ROMS; right).

Within the disease courses (ROMS and POMS, respectively), females with a relapsing onset are younger than males, but older for a progressive onset (32.1 *versus* 33.1, *p* < 0.001; 43.3. *versus* 42.3, *p* = 0.11). Whereas the differences are rather small within the various disease courses (1.1 years in ROMS and 1.0 year in POMS), the differences between the disease course (ROMS *versus* POMS) are larger (>10 years) and highly significant (*p* < 0.001). The female sex in itself has no significant influence on the age at the onset (*p* = 0.09). A significant effect can be seen if interactions between the course of the disease and the female sex are considered (*p* < 0.001). In addition to the higher age at diagnosis in patients with POMS (see Figure 1), the disease duration is longer and the mean Expanded Disability Status Scale (EDSS) score is higher. Patients with POMS retire at a later age, but after a much shorter period of illness than patients with ROMS, and to a greater extent (see Table 1). Considering the patients with relapsing onset, it was found that women left work significantly (*p* < 0.001) earlier (42.8 *versus* 44.2 years) and to a larger extent (28% *versus* 24%).

**Figure 2.**
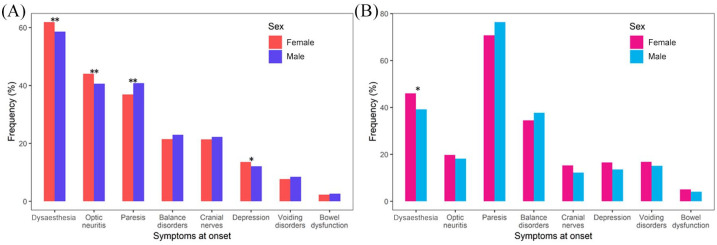
Frequency of initial symptoms in females and males, broken down by disease course [relapsing onset multiple sclerosis (A) and progressive onset multiple sclerosis (B)]. *p*-values: *p < 0.05, **p < 0.01

Optic neuritis and visual disturbances as first symptoms were more frequent in women than in men in both disease courses (ROMS *versus* POMS: women: 44% *versus* 20%, men: 41% *versus* 18%), but the difference was much less pronounced than between the disease courses (*p* < 0.001). Similarly, sensory deficits were more frequent (*p* < 0.001) in patients with ROMS than with POMS (females: 62% *versus* 46%, males: 59% *versus* 39%). Conversely, motor symptoms and cerebellar disorders were significantly (both: *p* < 0.001) more common in POMS (females: 71% *versus* 37% and 34% *versus* 21%, respectively, males: 76% *versus* 41% and 38% *versus* 23%, respectively). Statistically significant differences for women and men were found for motoric impairments (paresis) (*p* < 0.001) in patients with ROMS with male predominance, while women were more frequently affected by optic neuritis and sensory impairment (*p* < 0.001). In POMS patients there is a statistically significant difference between women and men for sensory disturbances as an initial symptom, with women being more affected than men (*p* = 0.039), whereas motor impairment was more frequently observed in men (*p* = 0.052); see Figure 2.

**Figure 3.**
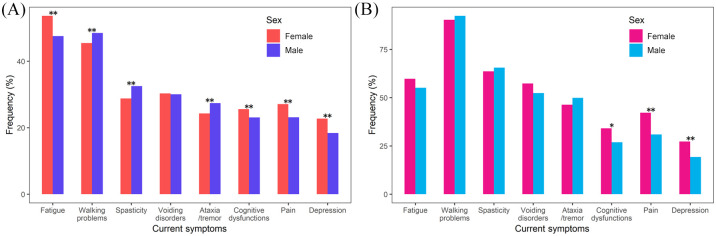
Frequency of current symptoms in females and males, broken down by disease course [relapsing onset multiple sclerosis (A) and progressive onset multiple sclerosis (B)]. *p*-values: *p < 0.05, **p < 0.01.

Figure 3 gives details of the current symptoms of the analysed patients. Gait problems, spasticity and ataxia are the most common symptoms in patients with POMS and are significantly (*p* < 0.001) more frequent compared with patients with ROMS. Symptoms with lower prevalence, including micturition problems (*p* < 0.001), pain (*p* < 0.001), constipation (*p* < 0.001) and dysarthria (*p* = 0.002), are significantly more common in patients with POMS than with ROMS.

Within ROMS patients, statistically significantly more women report fatigue, depression, pain (all: *p* < 0.001) and cognitive impairment (*p* = 0.001), while men are more often affected by spasticity and ataxia (*p* < 0.001). Regarding urogenital symptoms, sexual dysfunction is reported more frequently in men (*p* < 0.001), while micturition problems are reported more frequently in women.

In patients with POMS, women are statistically significantly more likely to experience impairment of cognition (*p* = 0.013) and depression (*p* = 0.002) and suffer more often from pain (*p* < 0.001). Men, on the other hand, are affected more frequently by sexual dysfunction (*p* = 0.002).

**Figure 4.**
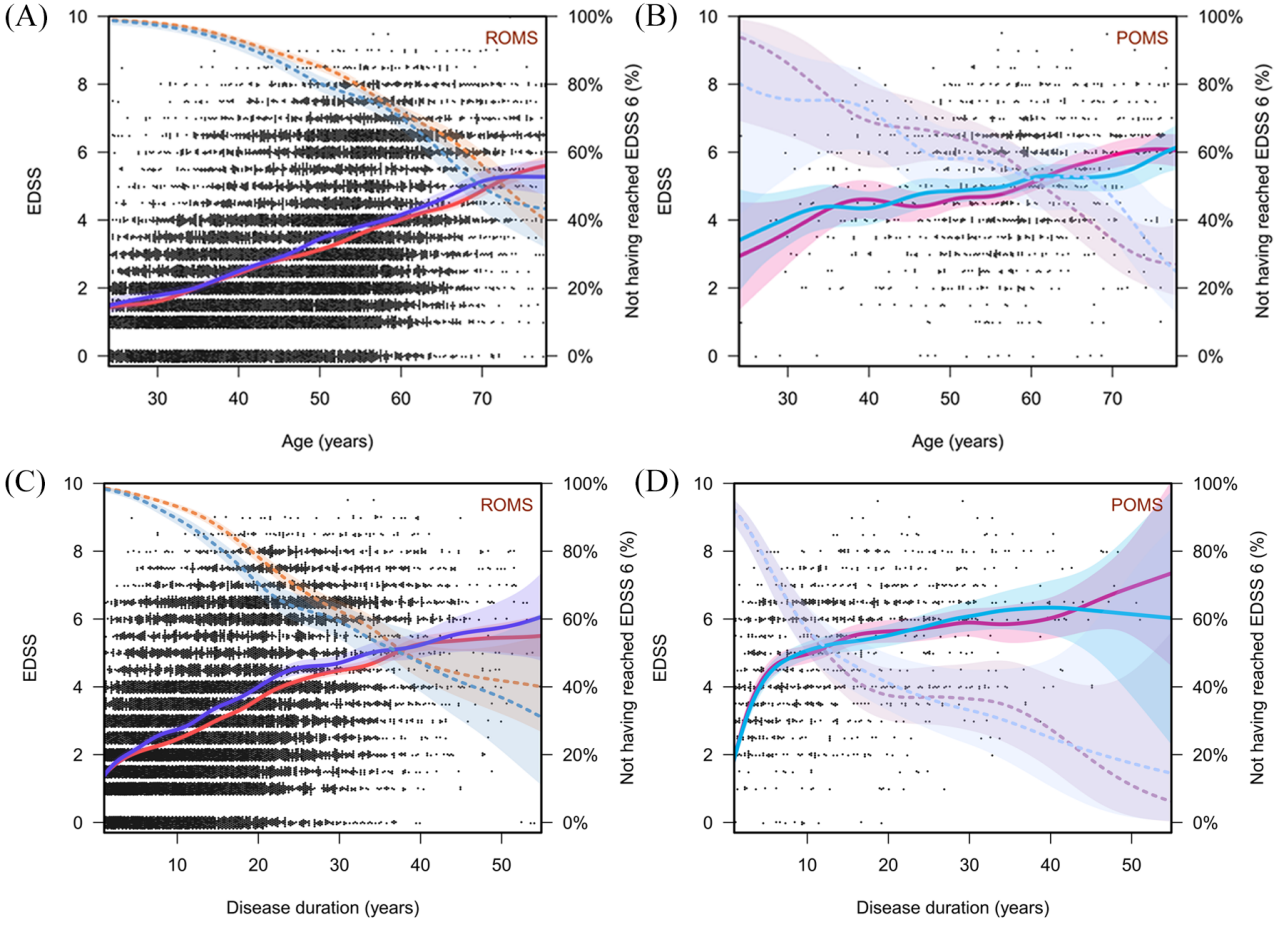
The temporal course of disability in women (red) and men (blue) in their respective disease courses and broken down by age and disease duration. (A) EDSS of patients with relapsing onset multiple sclerosis (ROMS) broken down by age. (B) EDSS of patients with progressive onset multiple sclerosis (POMS) broken down by age. (C) EDSS of patients with ROMS broken down by disease duration (years). (D) EDSS of patients with POMS broken down by disease duration (years). The solid line shows the proportion of patients with the corresponding EDSS (left axis). The dotted line shows the percentage of patients who have not reached an EDSS of 6 (right axis). EDSS, Expanded Disability Status Scale.

Figure 4 gives an overview of the development of disability in patients with ROMS and POMS grouped by women and men. The development of disability in patients is largely parallel for men and women in their respective courses, while patients with POMS reach EDSS 6 on average several years earlier. This is evident in terms of age and disease duration.

## Discussion

The main findings of our study are that we observed slight differences between men and women, in terms of both initial and current symptoms. Due to the high case numbers for ROMS, statistical significance is achieved for some initial symptoms such as motor and cerebellar deficits with male dominance, but the clinical relevance remains unclear. There is a higher prevalence of depression in women when symptoms first appear; this difference increases as the disease progresses and seems to be relevant already at an early stage. Women in particular are more affected in terms of neuropsychological and emotional symptoms such as fatigue, cognition, pain and, as already mentioned, depression for both disease courses. Pain perception was associated with depression and fatigue and it has been shown that women have a higher odds ratio than men to suffer from it.^16^

The number of patients differs significantly between men and women in our analysis. Therefore, we have carried out further analyses. We compared ROMS and POMS patients in 491 female and male patients with POMS in a 1:1 ratio according to disease progression, age at onset and duration of disease. This analysis confirmed the previously discussed results and is in accordance with the literature, where the age at onset of disease is 33.2 years for men and women in relapsing onset and 42.7 years (female) and 42.3 years (male) for patients with progressive onset.^1,12^ The striking findings with significantly more affected female patients with pain, depression, cognitive disorders are confirmed (*p* < 0.001).

The differences between the sexes are less pronounced than the differences between the courses of the disease.^17^ Some of these differences, such as significantly more frequent gait problems, spasticity, ataxia, fatigue, pain, micturition problems, sexual dysfunction and dysarthria in patients with POMS are probably due to the advanced stage of the disease (EDSS in POMS 5 *versus* 3 in ROMS). A new aspect of our analysis is that women with a relapsing onset leave work earlier and to a greater extent than men. This is surprising, since a more rapid disability progression and a faster progression of brain atrophy as well as a decrease in cognition has been described for male patients.^17–19^ However, in our study we showed that neuropsychological symptoms are more prevalent in women than in men even at an early disease stage. This discrepancy between the sexes was not seen in a study investigating depression^20^ and is not known for cognition impairment.^18^ In a small study, depression correlated with disability and negatively with employment status^21^ and may help to interpret our data. Interestingly, whereas females were more often affected by neuropsychological symptoms, males were more frequently affected by walking impairment, spasticity and ataxia. However, our data showed that the disability progression expressed by the EDSS was parallel for women and men.^17–19^

Nevertheless, we were able to confirm many of the differences described so far in our study. In accordance with the most recent reports, the sex ratio in our analysis is 2.6.^22^ The increased female-to-male ratio is mainly due to the ratio of 2.7 in patients with ROMS. About 93% of the patients analysed suffer from this disease course, while the sex ratio in patients with POMS is 1.3. One possible contributing factor for this different sex ratio may be the age at the onset of the disease, which for POMS is on average 10 years later in our analysis and in consistency with the literature.^1,12^ An evaluation of age dependency and the sex of patients showed that the sex ratio decreases with increasing age.^23^ In order to analyse this dependency in more detail, we studied the interactions between the sexes and the course of the disease. The course of the disease itself (*p* < 0.001), in contrast to the sex alone (*p* = 0.09), had a significant effect on age at onset. The interaction between sex and disease progression also showed a significant interaction (*p* = 0.009). No further interactions (for demographic and clinical data) between the course of the disease and the sex could be shown in the analyses. This could indicate that, in addition to the disease course, other factors, such as genetics or hormones,^19,24,25^ could be relevant and have an influence on the development and pathophysiology of the disease (e.g. inflammation,^14,26^ regeneration,^27,28^ including brain plasticity,^29–31^ and neurodegeneration).^32^ Pathophysiological differences between relapsing and progressive MS have been described. In relapsing–remitting MS patients (with significantly increased female-to-male ratio), the inflammatory component is the driver of disease activity, while in progressive patients (with an almost balanced female-to-male ratio) neurodegeneration is the most important.^1,33^ Histopathological studies showed that men harbour more smouldering lesions when aged 45–55 than women; however, above the age of 60 this difference balanced out; an effect of sex hormones was discussed.^34^ A predominance of the female sex is also found in other autoimmune diseases, such as systemic lupus erythematosus (SLE), in which the sex ratio changes with age in patients with the highest female-to-male ratio at childbearing age and decreases after menopause.^35^ Hormonal influences, and in particular oestrogen and its receptors, appear to influence pathogenesis and disease activity, although the underlying mechanisms are not understood.^36,37^ Hormonal effects on disease activity can also be discussed in humans, as the relapse rate in MS patients decreases during pregnancy but increases again after delivery.^38,39^ Interestingly, it is precisely this decrease in disease activity during pregnancy that is not observed in SLE patients.^40^ In animal models it was discussed whether testosterone is a protective factor for the development of an experimental autoimmune encephalomyelitis.^41^ These conflicting results again underline the distinctness of the individual autoimmune diseases and with different immune cell lines suggested to be responsible for disease progression.^42,43^ What has to be discussed is that sex hormones have a variety of effects on MS, as can be seen from the higher rate of disease in women, the effects of pregnancy on disease activity and those presented in our analysis. However, these differences are not fully understood. This is also due to the fact that there are a number of hormones, such as oestrogens, progesterone, androgens, prolactin, whose effects on the immune system are poorly understood, for example, oestrogens on the innate and adaptive immune system, and which are not understood under the influence and interaction of environmental factors.^44^

To study hormonal changes and effects on the disease, for example, after menopause, would be of great importance to be able to determine the influence of gender more precisely, but cannot be provided by our analysis. The different age for POMS and ROMS with different sex ratios can only be understood as a vague indicator of a correlation. The limitations of a registry are that data are not collected systematically as in a clinical trial. Neuropsychological symptoms have been evaluated by neuropsychological tests, specialist assessment by psychiatrists or clinical evaluation by the treating physician and our results must therefore be interpreted with knowledge of these limitations. However, these data reflect reality more than a laboratory situation.

## Conclusion

Our analysis shows that the differences in clinical presentation between men and women in MS persist across the different disease courses. The differences over the course of the disease are greater than the differences within the course of the disease between the sexes. However, depression, cognitive impairment and pain are more frequently reported in women across all disease courses. In addition, women with a relapsing onset of the disease leave work earlier. This is of great relevance and the reasons for that are unclear. Physicians should be aware of these differences and take appropriate measures (e.g. optimization of pain therapy, neuropsychological care, measures to promote employment).
